# Clinical outcome of a branch-first approach with a novel continuous whole-brain perfusion strategy for total arch surgery

**DOI:** 10.1186/s13019-024-02704-z

**Published:** 2024-04-16

**Authors:** Zezheng Zhao, Haitao Chi, Lei Chen, Junhui Wang, Cangsong Xiao

**Affiliations:** Department of Cardiovascular Surgery, The Sixth Medical Center, Chinese People’s Liberation Army General Hospital, Beijing, 100048 China

**Keywords:** Total arch surgery, Cerebral protection, Novel whole-brain perfusion, Unilateral antegrade cerebral perfusion

## Abstract

**Background:**

Cerebral protection strategies have been investigated since the introduction of aortic arch surgery and have been modified over the centuries. However, the cerebral protective effects of unilateral and bilateral antegrade cerebral perfusion are similar, with opportunities for further improvement.

**Methods:**

A total of 30 patients who underwent total arch surgery were enrolled in this study. Patients were assigned to the novel continuous whole-brain or unilateral antegrade cerebral perfusion group according to the cerebral perfusion technique used. Preoperative clinical data and 1-year postoperative follow-up data were collected and analyzed.

**Results:**

There were no significant differences between the two groups in terms of the incidence of permanent neurological deficit, mortality, or therapeutic efficacy. However, the incidence of temporary neurological dysfunction in the novel whole-brain perfusion group was significantly lower than that in the unilateral antegrade cerebral perfusion group.

**Conclusions:**

In this study, the branch-first approach with a novel whole-brain perfusion strategy had no obvious disadvantages compared with unilateral antegrade cerebral perfusion in terms of cerebral protection and surgical safety. These findings suggest that this new technique is feasible and has application value for total arch surgery.

## Introduction

Cerebral protection is very important in aortic arch (AOA) surgery. Cerebral protection strategies have been investigated since the introduction of aortic arch surgery and have been modified over the centuries [1–3]. The cerebral protective effects of unilateral antegrade cerebral perfusion (u-ACP) and bilateral antegrade cerebral perfusion (b-ACP) are similar, with room for further improvement [4–6]. Typically, total arch replacement (TAR) is performed under selective u-ACP or b-ACP with hypothermic circulatory arrest (HCA). Reconstruction of supraaortic vessels (SAVs) is usually performed under the above conditions and may result in central nervous system (CNS) complications [7–9]. To address this, an increasing number of cardiac surgeons are applying new surgical techniques, such as minimally invasive surgery and whole-brain perfusion (WBP). Our previous studies confirmed the feasibility and safety of SAVs reconstruction combined with moderate hypothermic b-ACP [10, 11]. On this basis, we treated 12 patients with the branch-first approach with a novel WBP (n-WBP) strategy, and treatment was successful in all patients.

To confirm the safety, advantages, and early therapeutic effects of this new technique, we retrospectively analyzed the clinical data of 30 patients who underwent total arch surgery using u-ACP or n-WBP at our center from January 2017 to June 2020.

## Materials and methods

### Patients

This was a single-center retrospective cohort study. The inclusion criteria were as follows: (1) Preoperative computed tomography angiography (CTA) of the aorta showed that the intimal tear involved both the greater and minor curvature of the arch. (2) TAR surgery was performed. (3) u-ACP or n-WBP was performed. (4) The patient did not have severe neurologic disorders. (5) The patient did not have severe dysfunction of the liver, kidney or other organs. The exclusion criteria were as follows: (1) Preoperative CTA of the aorta showed that the intimal tear involved only the minor curvature of the arch. (2) Hemiarch replacement surgery was performed. (3) b-ACP was performed. (4) Only ascending aorta (AAO) or aortic root replacement/repair surgery was performed. (5) Coronary, mitral or tricuspid valve operation was performed simultaneously. (6) The patient had severe neurologic disorders or dysfunction of other organs. (7) Severe complications such as aortic rupture or shock occurred before the operation. (8) Clinical data were lacking. A total of 144 patients underwent AOA surgery at our center between January 2017 and June 2020. Thirty patients were eventually included in the study.

Of the 30 patients (aged 22–74 years), 25 were male and 5 were female. All patients presented with different degrees of chest and back pain. All patients underwent CTA of the aorta and were diagnosed with acute type A aortic dissection. Transthoracic echocardiography (TTE) was routinely performed to assess cardiac function and the condition of the cardiac chamber and valve. The patients had no serious complications, such as aortic rupture, cardiac tamponade or organ malperfusion. None of the patients had experienced a CNS disease, such as cerebral infarction, cerebral hemorrhage, brain tumor, epilepsy or Parkinson’s disease, within the previous months. CTA of the aorta showed that the intimal tear involved both the greater and minor curvatures of the arch. Therefore, we implemented TAR for all patients. The open thoracic approach was selected as the surgical approach for all patients. The basic characteristics of the patients are shown in Table 1.

**Table 1:** Basic characteristics of patients in each group

Items	u-ACP group (*N* = 18)	n-WBP group (*N* = 12)	t/Z-values	*P*-values
Male (n, %)	15 (83.33%)	10 (83.33%)	——	1.000
Female (n, %)	3 (16.67%)	2 (16.67%)	——	1.000
Age (years, mean ± SD)	42.83 ± 9.55	45.92 ± 14.98	-0.69	0.495
BMI (Kg/m^2^, mean ± SD)	27.92 ± 3.78	25.79 ± 3.18	1.61	0.120
Hypertension (n, %)	11 (61.11%)	6 (50.00%)	——	0.711
Smoking (n, %)	5 (27.78%)	2 (16.67%)	——	0.669
Alcohol consumption (n, %)	3 (16.67%)	2 (16.67%)	——	1.000
Marfan syndrome (n, %)	1 (5.56%)	1 (5.56%)	——	1.000
Erythrocyte (10^12^/L, mean ± SD)	4.10 ± 0.80	3.54 ± 0.73	1.95	0.061
Hemoglobin (g/L, mean ± SD)	125.78 ± 24.87	108.92 ± 23.04	1.87	0.072
Platelet (10^9^/L, mean ± SD)	175.22 ± 66.72	164.08 ± 85.76	0.40	0.692
APTT (S, mean ± SD)	42.61 ± 10.57	47.88 ± 15.96	-1.09	0.284
EF [%, median (IQR)]	65.00 (5.50)	64.50 (5.75)	-0.30	0.761
AAOR + TAR + DAOSG (n, %)	16 (88.89%)	11 (91.67%)	——	1.000
AAOR + TAR (n, %)	2 (11.11%)	1 (5.56%)	——	1.000
David (n, %)	1 (5.56%)	1 (5.56%)	——	1.000
AVP (n, %)	12 (66.67%)	7 (58.33%)	——	0.712

Note: Continuous measurement data, t/Z-values and count data percentage are rounded to two decimal places. *P*-values are rounded to three decimal places. BMI: Body Mass Index; APTT: Activated partial thromboplastin time; EF: ejection fraction; AAOR: AAO replacement; TAR: total arch replacement; DAOSG: descending aorta stent graft; AVP: aortic valvuloplasty.

The study was approved by the Ethics Committee of Chinese People’s Liberation Army General Hospital (No. 2019-JCJQ-ZD-195-00). Informed consent was obtained from all the participants in the study.

### Study groups

This was a retrospective cohort study involving 30 patients. Of the 30 patients, 12 underwent the branch-first approach with a n-WBP strategy and were assigned to the n-WBP group; the remaining 18 underwent u-ACP and were assigned to the u-ACP group.

Patients in the n-WBP group received reconstruction of the SAVs through heart ejection without cardiopulmonary bypass (CPB). There were 10 males and 2 females among the 12 patients. All 12 patients underwent AAO replacement (AAOR), TAR and descending aorta stent graft (DAOSG) surgery. One patient underwent aortic root replacement using the David procedure. Seven patients underwent aortic valvuloplasty (AVP). Patients in the u-ACP group underwent reconstruction of the SAVs through CPB. There were 15 males and 3 females among the 18 patients. Sixteen patients underwent AAOR, TAR and DAOSG surgery. Two patients underwent AAOR and TAR. Twelve patients underwent AVP.

### Surgical technique

The patient was placed in the supine position. Single-cavity tracheal intubation was performed after the induction of general anesthesia. Bilateral radial artery pressure, dorsal foot artery pressure and cerebral perfusion flow were measured. Transcranial infrared techniques were used to monitor cerebral oxygenation. A Swan-Ganz catheter and 7 F deep venous tubing were inserted via the right internal jugular vein to monitor central venous pressure.

**Fig. 1:**
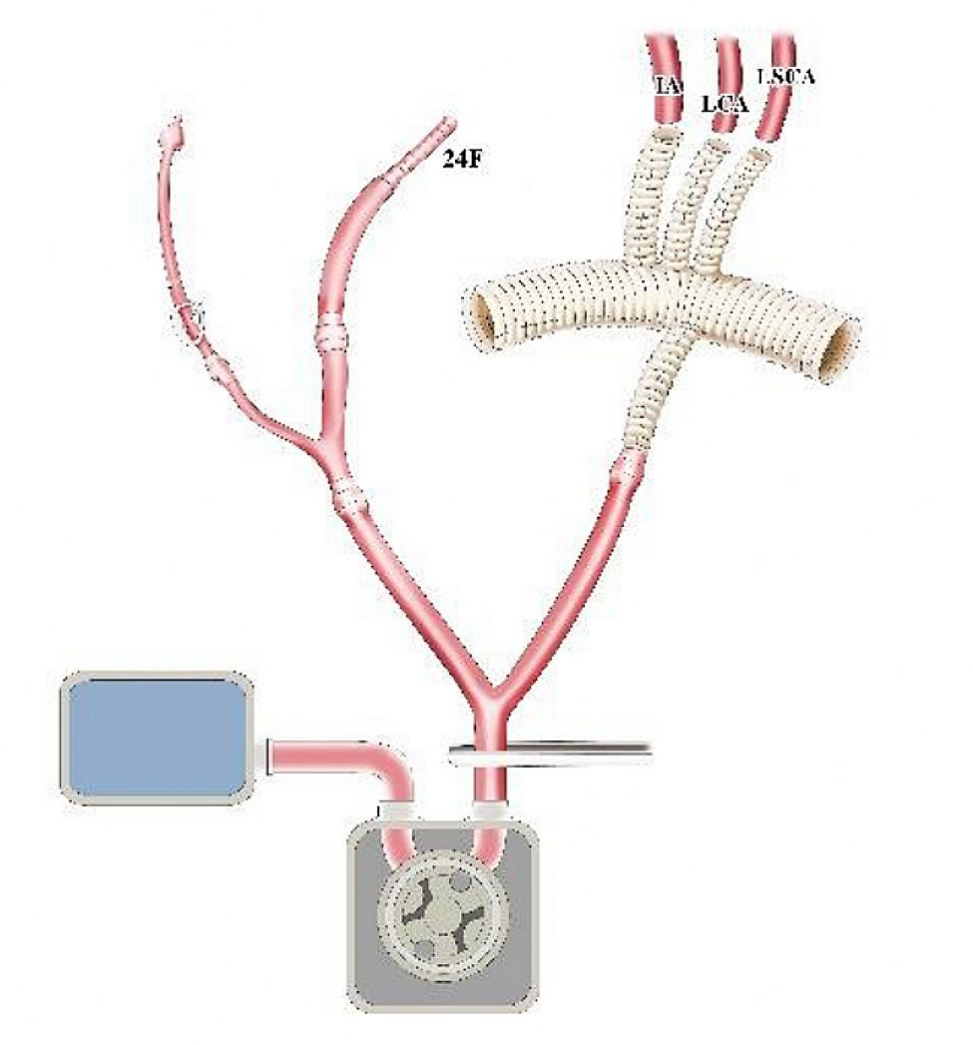
Schematic diagram of single-pump and double-tube connection for CPB

**Fig. 2:**
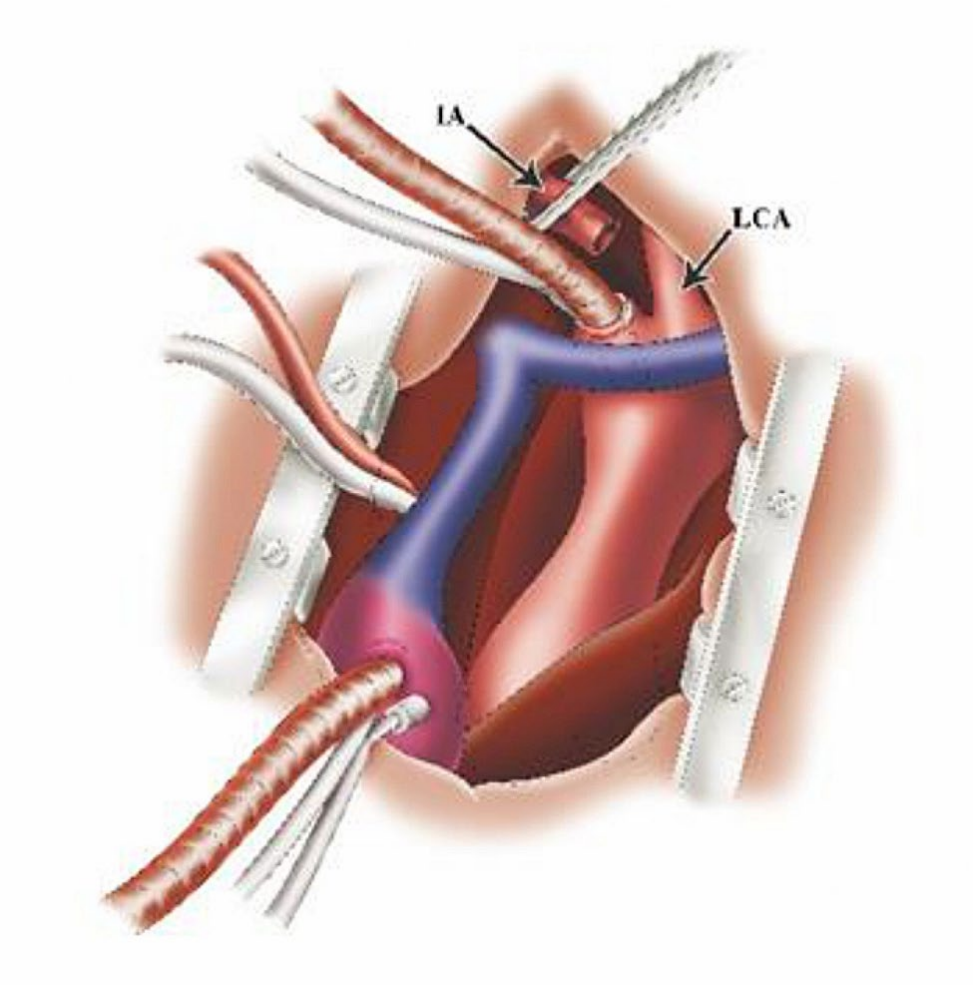
Schematic diagram of aortic cannulation

After sternotomy, the innominate artery (IA), left common carotid artery (LCCA) and left subclavian artery (LSCA) were adequately freed. Y-type conversion tubing was connected to one output end of the Y-type arterial perfusion tubing. The two output ends of the Y-type conversion tubing were respectively connected to 24 F and 14 F tubing (Fig. 1). A pipe plier was used to clamp the output end of the Y-type arterial perfusion tubing. The 14 F tubing was also clamped. Another output end of the Y-type arterial perfusion tubing was connected to the perfusion branch of the four-branch vascular graft (f-b-VG) (Medtronic 28 Dacron). The root of the Y-type arterial perfusion tubing was then clamped (Fig. 1). A venous cannula was inserted through the right atrium to establish the CPB circuit. A surrounding purse-string suture was placed at the root of the IA. After cutting the anterior wall of the IA (with the partial posterior wall preserved) and identifying the true and false lumen, the 24 F tubing was inserted into true lumen of aortic arch through the incision (Fig. 2). 

**Fig. 3:**
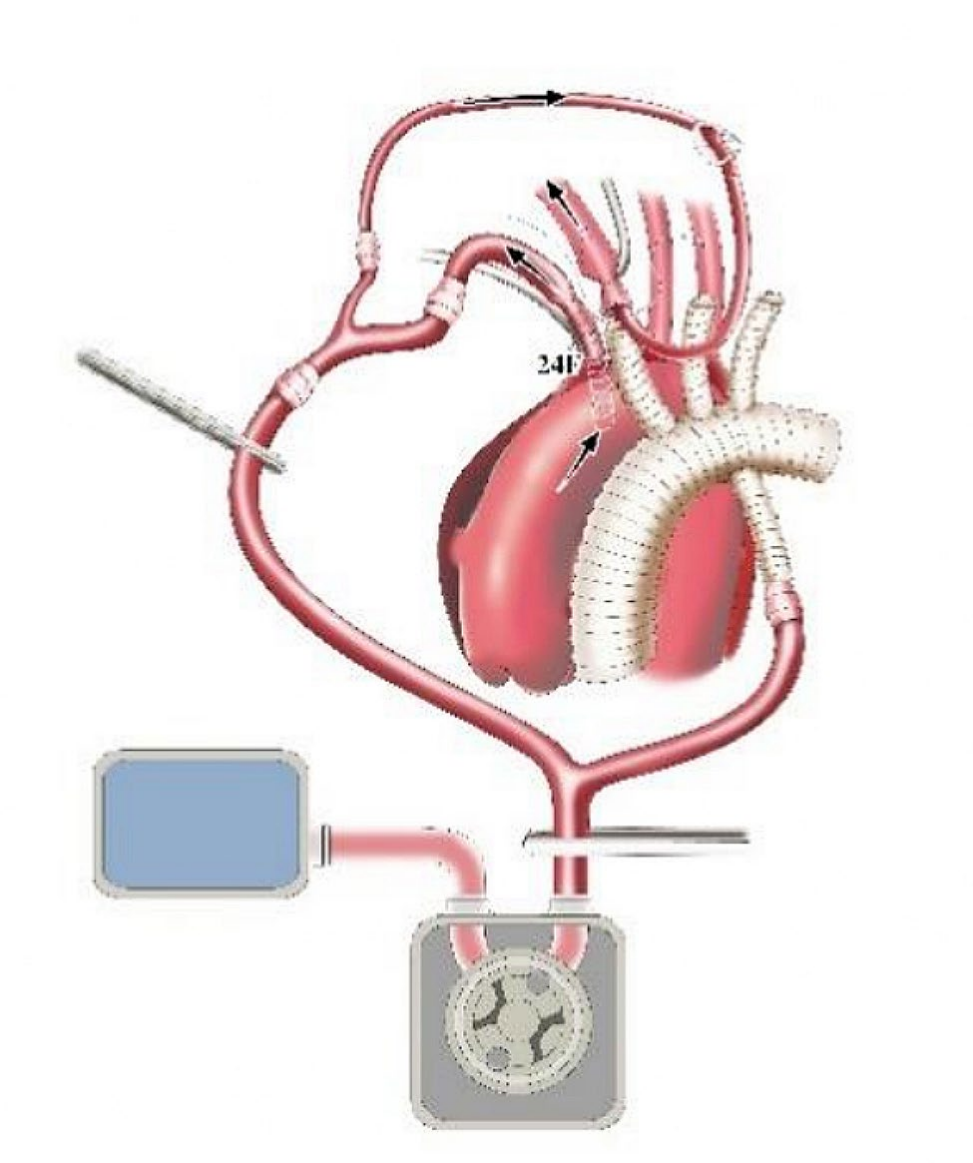
Schematic diagram of anastomosing the first branch of SAVs

**Fig. 4:**
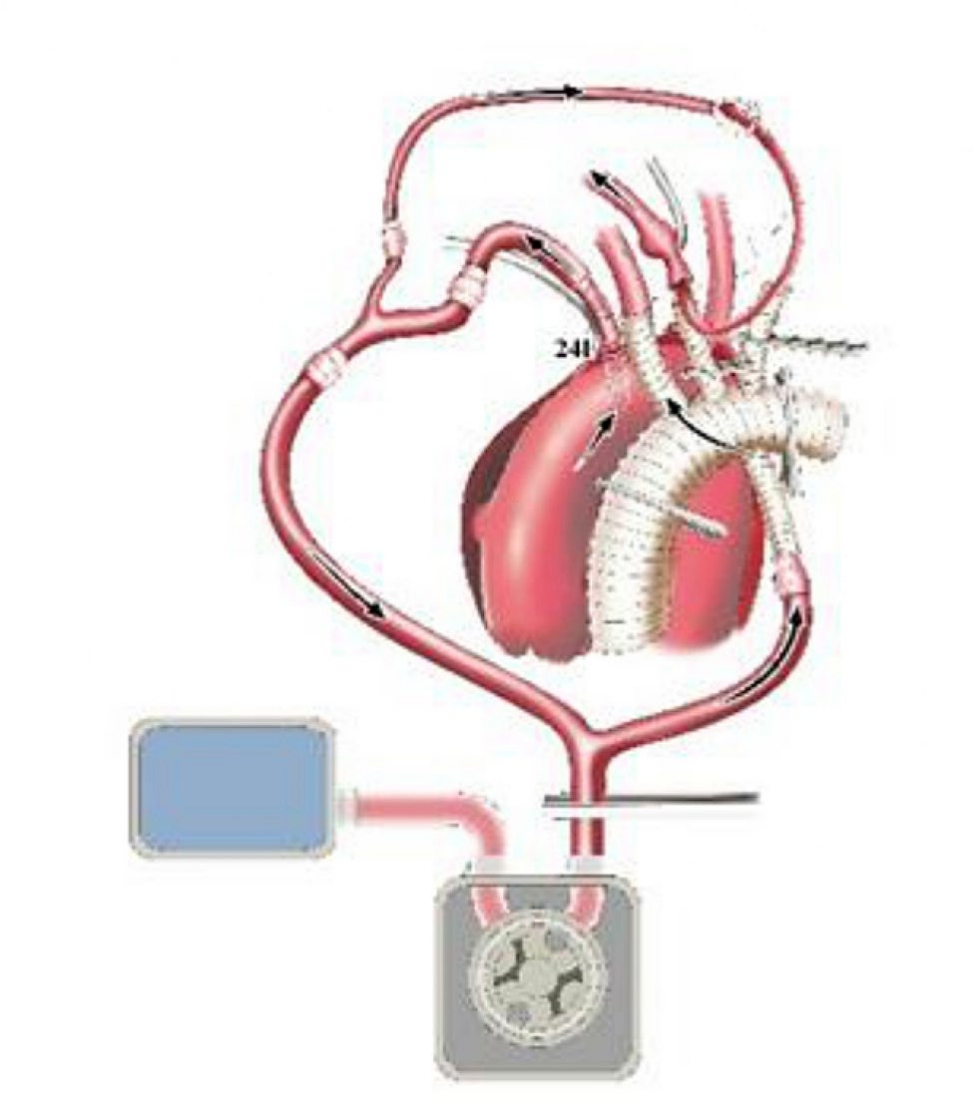
Schematic diagram of anastomosing the second branch of SAVs

**Fig. 5:**
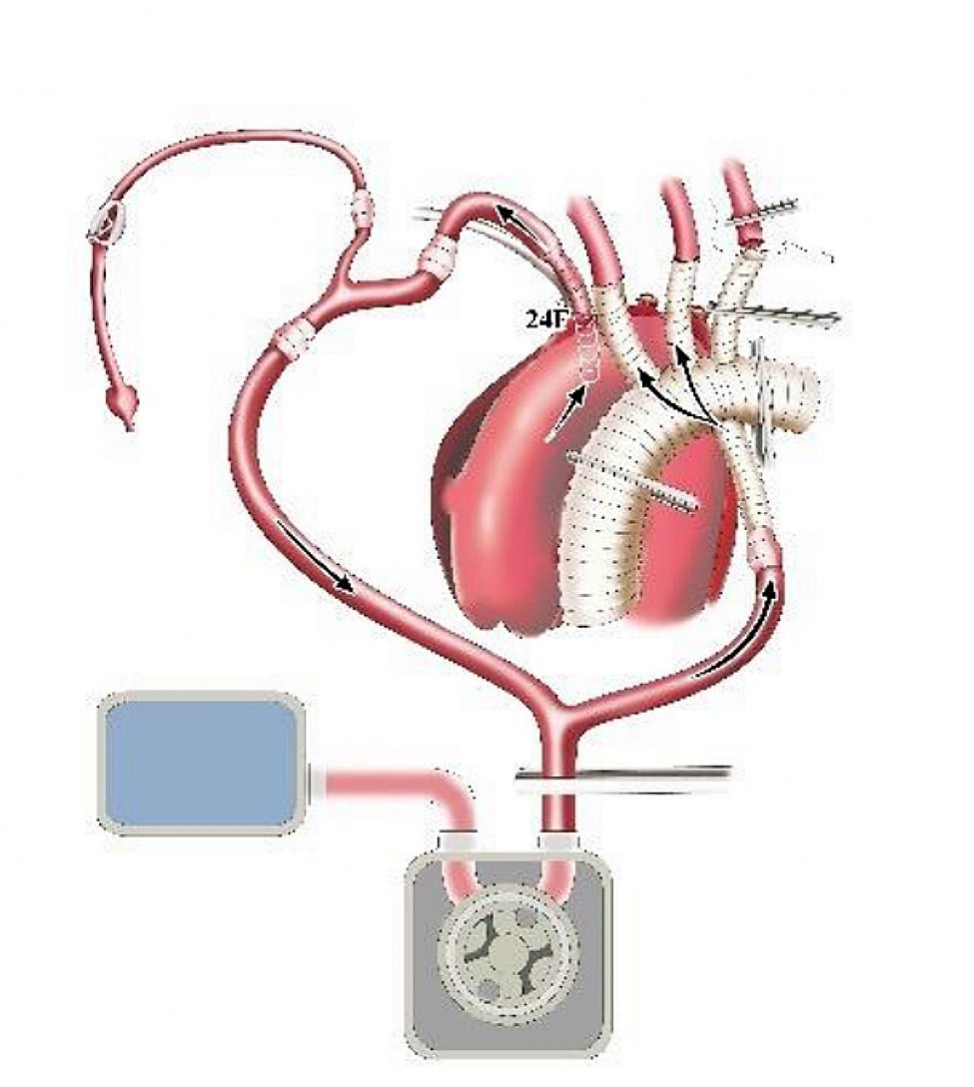
Schematic diagram of anastomosing the third branch of SAVs

The 14 F tubing was inserted into the distal lumen of IA and opened to form a cerebral perfusion circuit and its blood flow was resumed. Immediately after this maneuver, without CPB initiation, the first branch (10 mm) of the f-b-VG was anastomosed to the IA during which its blood flow was continued, aiming to eliminate the potential risk of cerebral ischemia associated with traditional debranching technique in which blood flow of the transected distal IA was totally interrupted (Fig. 3). The 14 F tubing was clamped and removed when the anastomosis was completed. The sutures were tied. The two remaining branches and ends of the vascular graft (VG) were clamped. The output end of the Y-type arterial perfusion tubing was opened (with continued root clamping) to restore the blood supply to the IA (Fig. 4). Reconstruction of the other SAVs was performed as described above (Figs. 4–5), except for the 14 F cannula was not required to perfuse the distal LSCA. All of the above operations were performed without CPB.

**Fig. 6:**
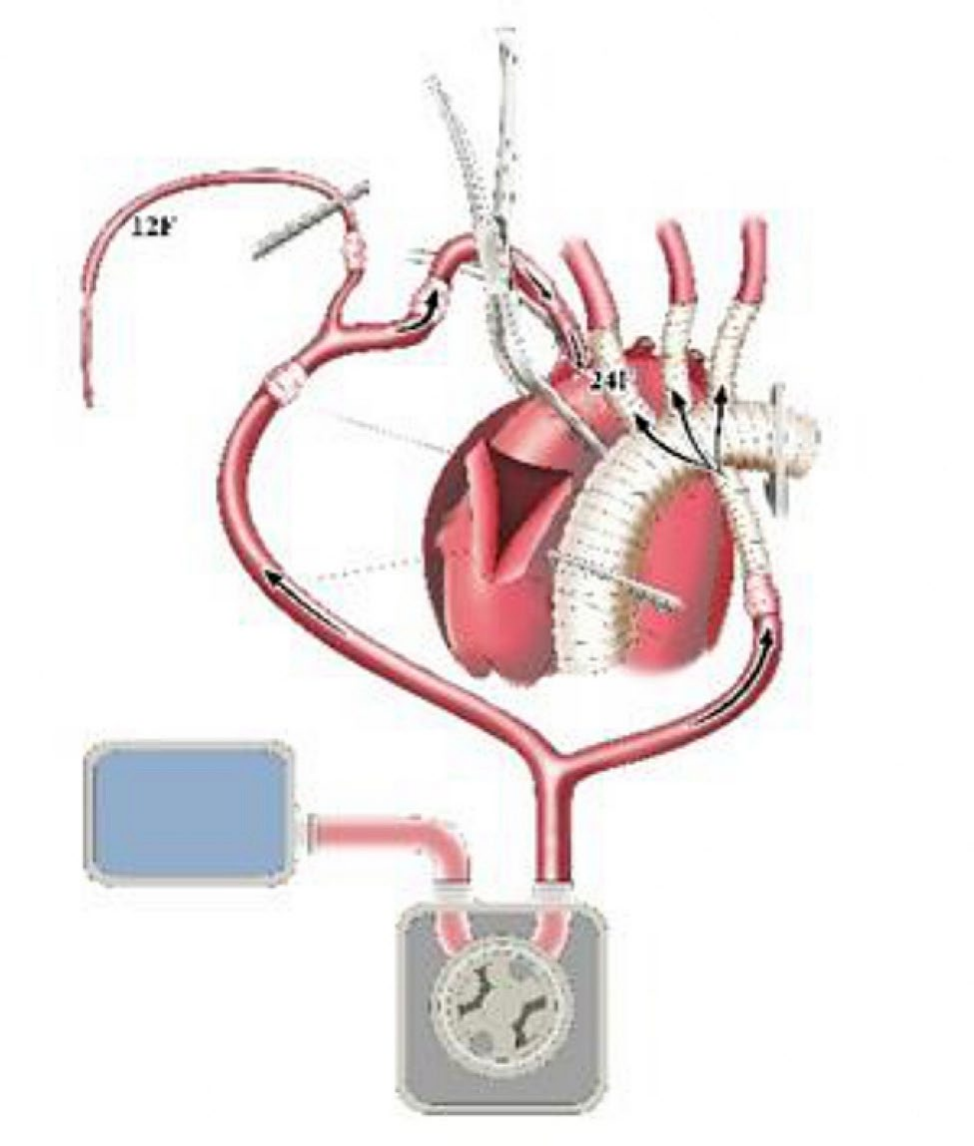
Schematic diagram of cutting aortic wall

CPB was initiated to cool the entire body after completion of SAV anastomosis. Perfusion flow during CPB was appropriately controlled through monitoring the radial and dorsal foot artery pressures so that the perfusion pressures of the upper and lower body were as equal as possible. The AAO was clamped and incised. HTK cardioplegia was administered via left and right coronary ostia. The aortic wall was transected 1 cm above the sinotubular junction (Fig. 6). Aortic root repair or replacement was performed according to its pathology.

**Fig. 7:**
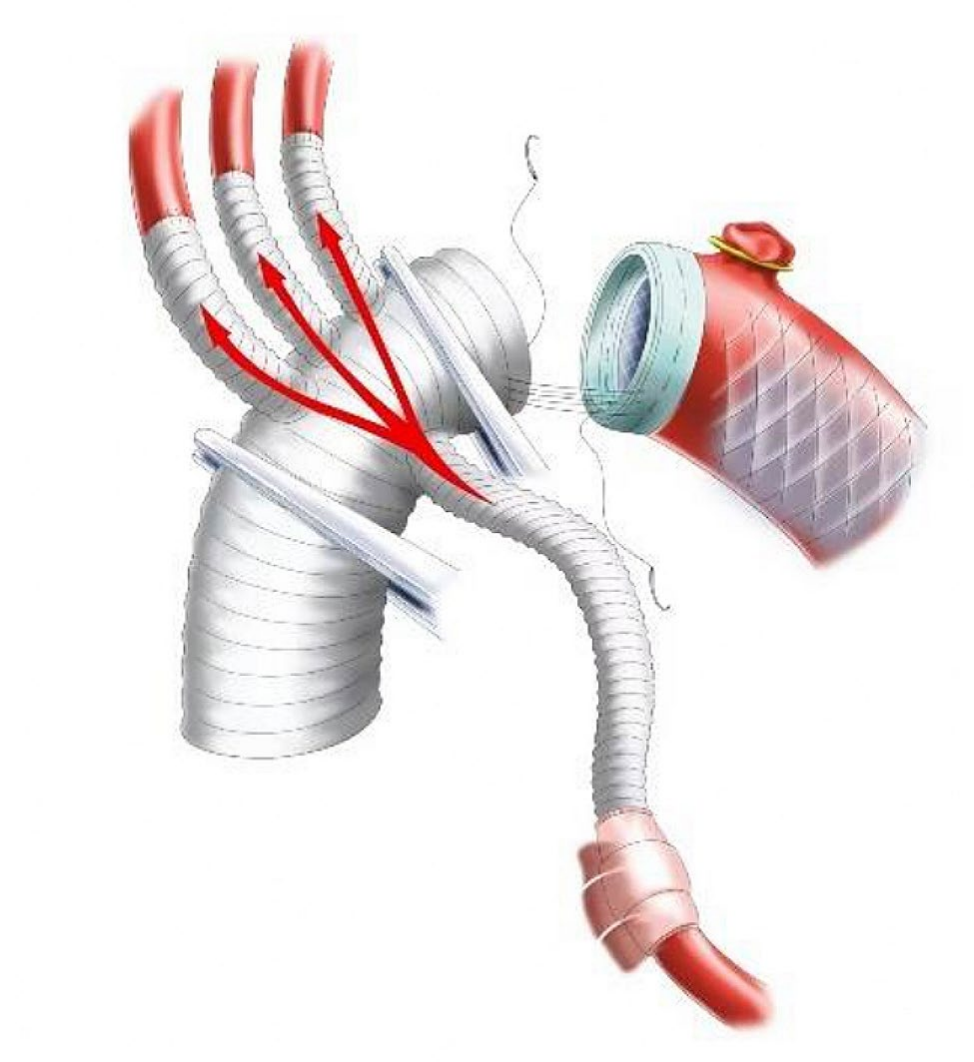
Schematic diagram of WBP during anastomosing the distal end of f-b-VG

**Fig. 8:**
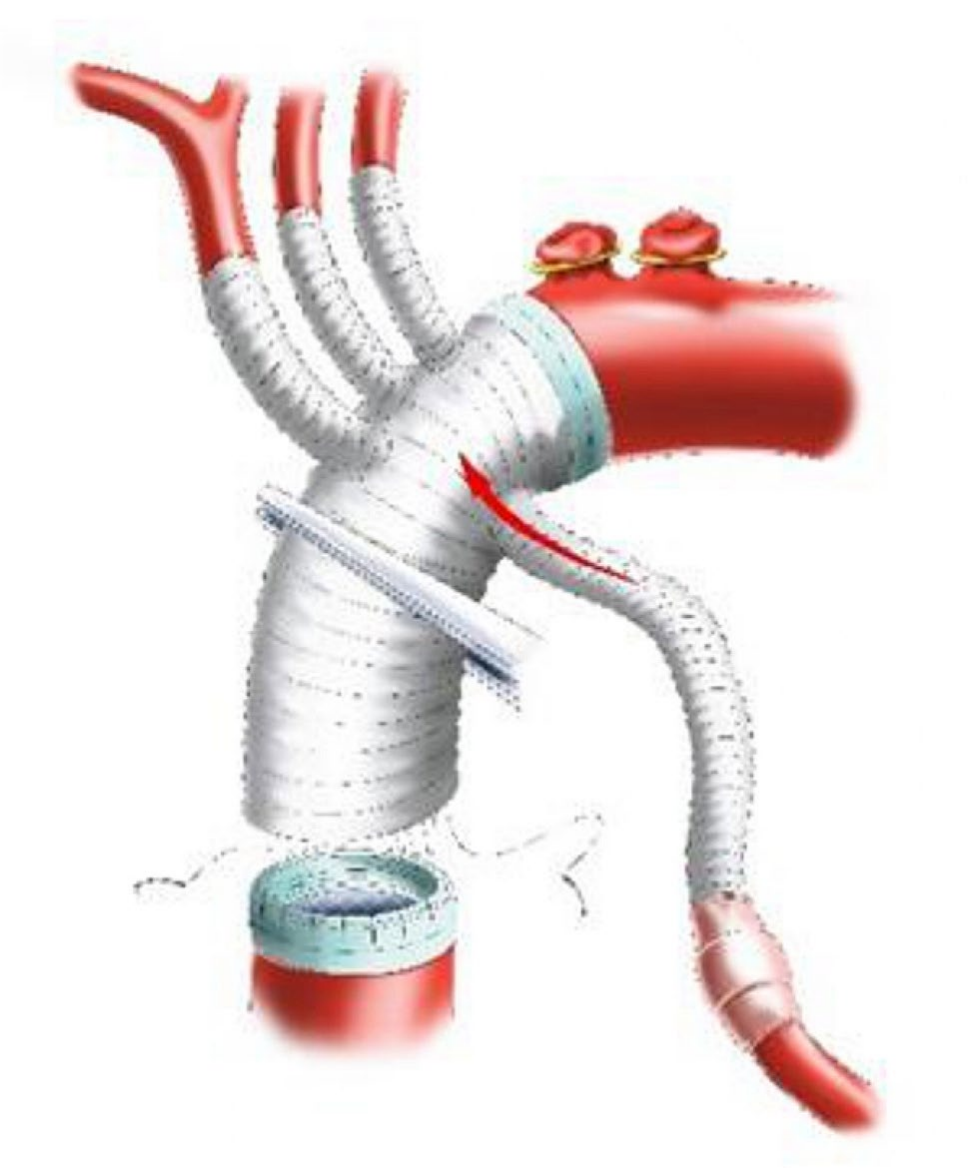
Schematic diagram of the systemic perfusion restoration

Lower body HCA was performed when the bladder temperature decreased to approximately 30 °C. Perfusion, namely WBP, was continued in the brain and upper body, maintaining a radial artery pressure of approximately 30 mmHg, a perfusion flow rate of 5–8 ml/min/kg and a cerebral oxygenation of approximately 60%. Preoperative CTA showed no reentrance in the proximal descending aorta (DAO) in this 30 patients. Based on the diameter of the DAO measured on preoperative CTA, an appropriately sized stent graft (SG) (Medtronic 28 Nitinol) was selected. The AOA was transected between the LCCA and LSCA or between the IA and LCCA, and the selected SG was carefully implanted into the true lumen of the DAO. The SG suture sleeve and distal aortic stump were trimmed so that the end of the SG was flush with the distal aortic stump (the SG was propped up to open the anastomosis of the aorta). A felt strip was added to the outside of the aortic wall, which was anastomosed to the distal end of the f-b-VG (Fig. 7). Systemic perfusion was subsequently restored, and rewarming was initiated via the perfused side branch of the f-b-VG (Fig. 8). Finally, the proximal end of the f-b-VG was anastomosed to the proximal aortic stump. After completion of anastomosis, aortic cross-clamping (ACC) was ceased to restore coronary blood supply.

### Data collection

Basic patient and surgical information was collected from our center’s data management system. This information included age, BMI, hypertension status, smoking status, alcohol consumption and surgical methods (Table 1). In addition, we collected intraoperative and postoperative data (Table 2). The intraoperative data included CPB, ACC, HCA duration, cerebral oxygenation during cerebral perfusion, the amount of erythrocytes and plasma transfused, and minimum nasopharyngeal temperature. The postoperative data included ventilator-assisted respiration time (VART), length of stay in the cardiac intensive care unit (CICU), the occurrence of acute renal failure (ARF) requiring continuous renal replacement therapy and mortality.

**Table 2:** Intraoperative and postoperative data of patients in each group

Items	u-ACP group (*N* = 18)	n-WBP group (*N* = 12)	t/Z-values	*P*-values
Intraoperative information				
Cerebral oxygenation during cerebral perfusion (%, mean ± SD)	64.94 ± 1.73	65.58 ± 1.17	-1.12	0.273
Minimum nasopharyngeal temperature [℃, median (IQR)]	27.10 (2.20)	26.30 (1.10)	-0.89	0.373
CPB duration (min, mean ± SD)	234.83 ± 36.29	196.17 ± 12.06	-3.37	< 0.001
ACC duration (min, mean ± SD)	163.67 ± 31.80	133.33 ± 6.30	-3.09	0.002
HCA duration (min, mean ± SD)	44.44 ± 3.65	27.08 ± 3.20	13.38	< 0.001
Erythrocyte (U, mean ± SD)	4.17 ± 1.47	2.92 ± 1.78	2.10	0.045
Plasma (U, mean ± SD)	8.83 ± 1.46	6.66 ± 1.09	4.41	< 0.001
Platelet [U, median (IQR)]	2.00 (0.00)	2.00 (0.75)	-1.67	0.960
Postoperative information				
Operative area drainage [mL, median (IQR)]	435.00 (97.50)	427.50 (98.75)	-0.81	0.419
VART (h, mean ± SD)	59.97 ± 32.77	28.38 ± 12.77	-2.39	0.017
CICU stay [h, median (IQR)]	108.00 (78.00)	72.00 (66.00)	-2.08	0.038
ARF (undergoing CRRT) (n, %)	1 (5.56%)	1 (8.33%)	——	1.000
TND (n, %)	9 (50.00%)	1 (8.33%)	——	0.024
PND (n, %)	0	0	——	——
Mortality (n, %)	0	0	——	——

Note: Continuous measurement data, t/Z-values and count data percentage are rounded to two decimal places. *P*-values are rounded to three decimal places. CRRT: continuous renal replacement therapy; TND: temporary neurological dysfunction; PND: permanent neurological deficit.

To evaluate the cerebral protective effect of n-WBP, we collected data on postoperative neurological complications, such as permanent neurological deficit (PND) and temporary neurological dysfunction (TND). PND was defined as focal or global neurologic deficits with corresponding abnormalities on cranial CT images that persisted after discharge, including cerebral infarction and hemorrhage. TND was defined as temporary or nonfocal neurologic dysfunction associated with typically normal cranial CT findings and included manifestations of psychotic disorders such as epilepsy, delirium and agitation, that disappeared before discharge.

### Follow-up

To confirm early surgical efficacy in the 30 patients, we followed up all patients by telephone and WeChat, mainly to assess symptoms and adverse events in the first year after surgery. The symptoms included thoracic and back pain, chest tightness, palpitations, and fatigue. The adverse events included dilatation of the arch and DAO, secondary surgery and severe CNS disorders such as cerebral infarction and hemorrhage. The presence of these events was assessed by CTA and TTE at 1 year after surgery.

### Quality control

All 30 patients underwent surgery by the same experienced team. To assess patients who developed CNS complications after surgery, the same experienced neurologist reviewed the symptoms and CT images to ensure diagnostic accuracy. To keep the collected data as accurate and comprehensive as possible, two professionals collected both perioperative and follow-up data. A third professional analyzed the data and made the final determination when there was a disagreement.

### Statistical analysis

The collected data were statistically analyzed and plotted using SPSS 27.0 (IBM Corporation, Armonk, NY, USA) and GraphPad Prism 9.0 (GraphPad Software, La Jolla, CA, USA), respectively. The Kolmogorov–Smirnov test was used to assess the goodness of fit of the data to a normal distribution. Continuous variables conforming to a normal distribution are expressed as the mean ± standard deviation, and nonnormally distributed continuous variables are expressed as the median and interquartile range. Student’s T test was performed to analyze differences in normally distributed continuous variables between two groups, and the Mann–Whitney U test was performed for continuous variables that were not normally distributed. Count information for the two groups is expressed as the number of patients and percentage. Differences in count data between the two groups were analyzed using Pearson’s chi-square test or Fisher’s exact test. *P* < 0.05 indicated statistical significance.

## Results

There were no significant differences in baseline data (including the surgical approach) between the two groups (Table 1), and the intraoperative and postoperative data were comparable.

### Comparison of intraoperative data

**Fig. 9:**
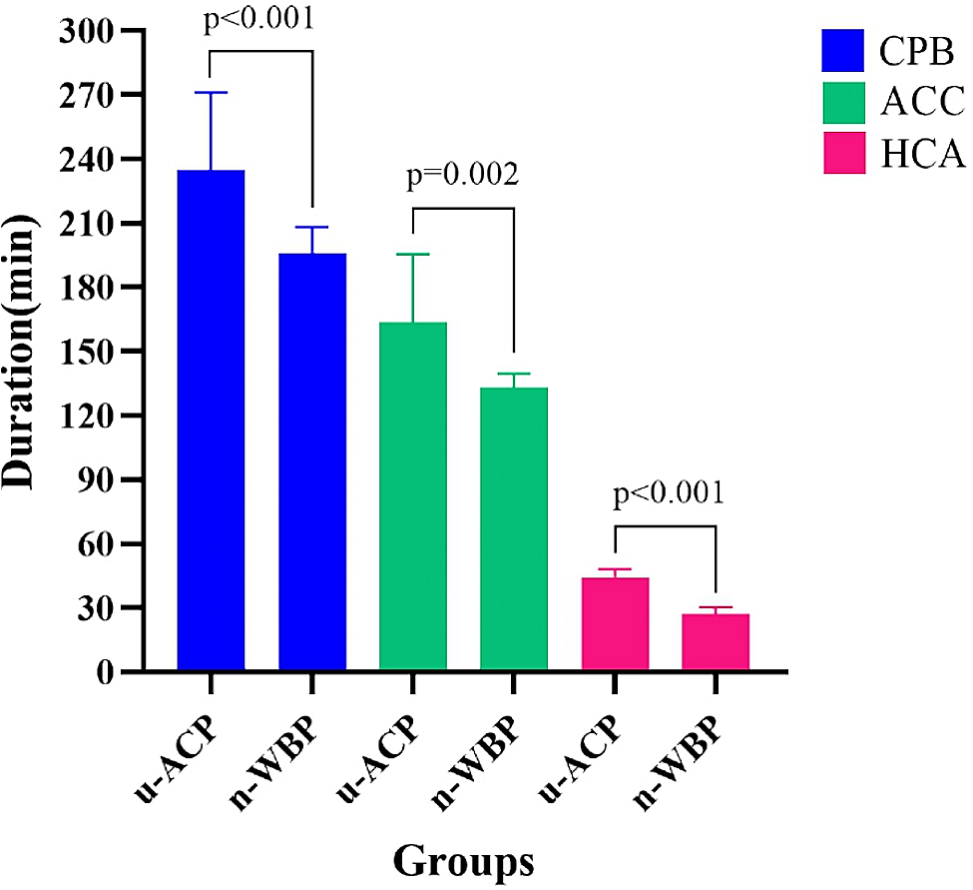
Column diagram of CPB, ACC and HCA durations

**Fig. 10:**
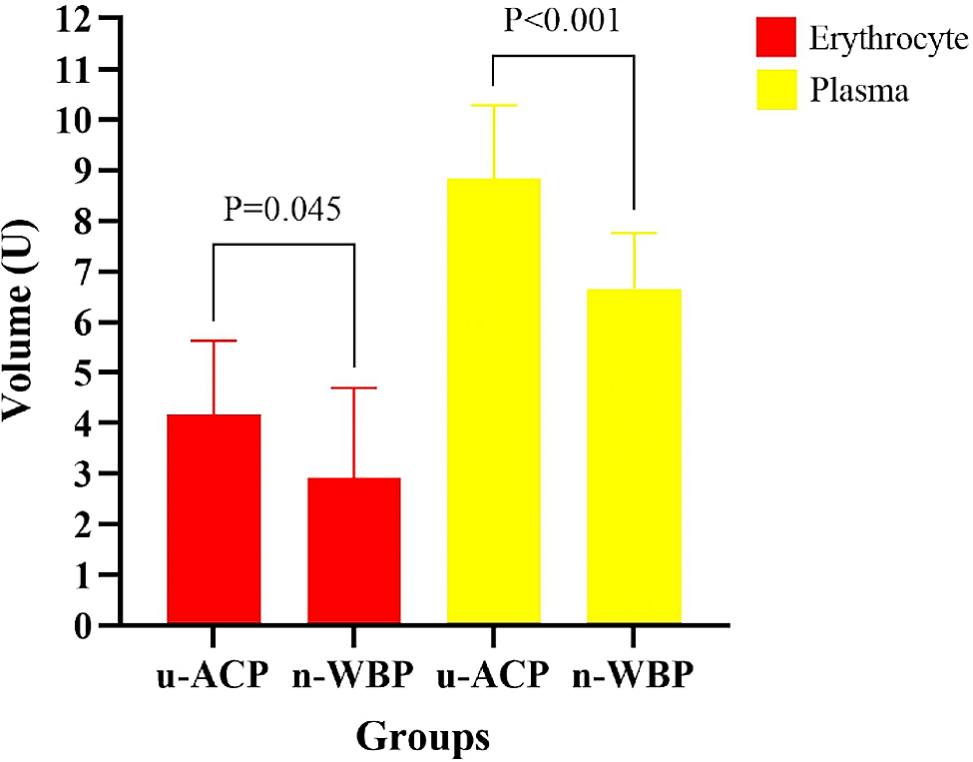
Column diagram of erythrocyte and plasma

There were no significant differences in cerebral oxygenation or minimum nasopharyngeal temperature between the two groups (Table 2). Compared with those in the u-ACP group, patients in the n-WBP group had a shorter CPB duration [196.17 ± 12.06 vs. 234.83 ± 36.29 min, *P* < 0.001], ACC duration [133.33 ± 6.30 vs. 163.67 ± 31.80 min, *P* = 0.002] and HCA duration [27.08 ± 3.20 vs. 44.44 ± 3.65 min, *P* < 0.001] (Fig. 9). In addition, fewer erythrocytes [2.92 ± 1.78 vs. 4.17 ± 1.47 U, *P* = 0.045] and plasma [6.66 ± 1.09 vs. 8.83 ± 1.46 U, *P* < 0.001] were administered in the n-WBP group (Fig. 10).

### Comparison of postoperative data

**Fig. 11:**
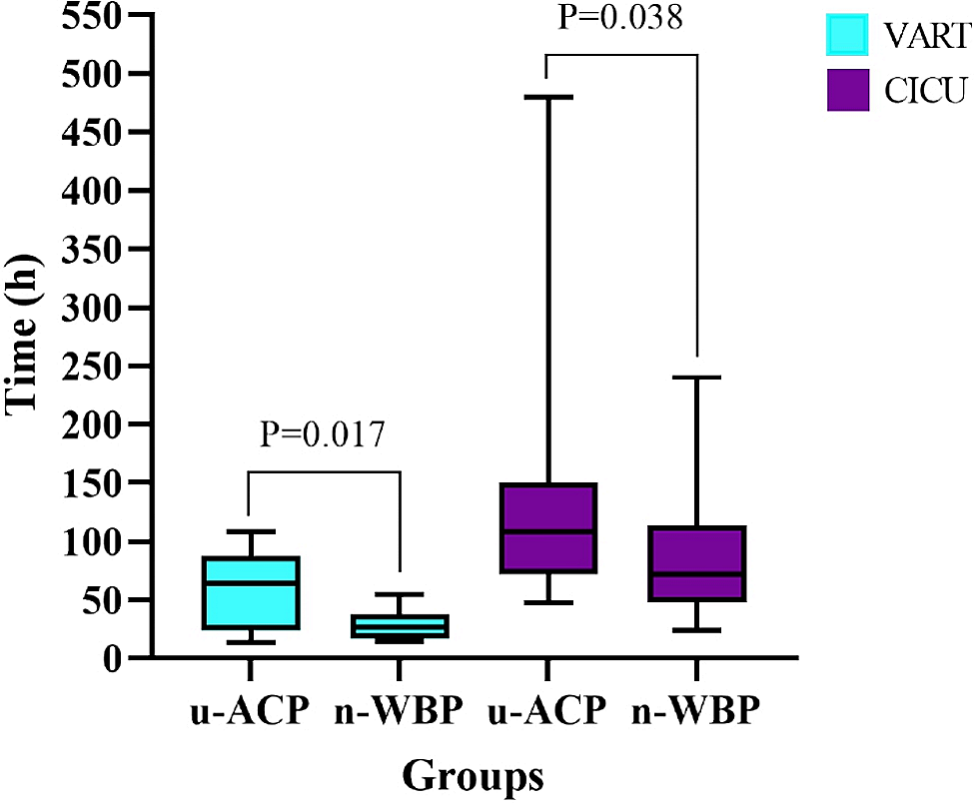
Box diagram of VART and CICU stays

**Fig. 12:**
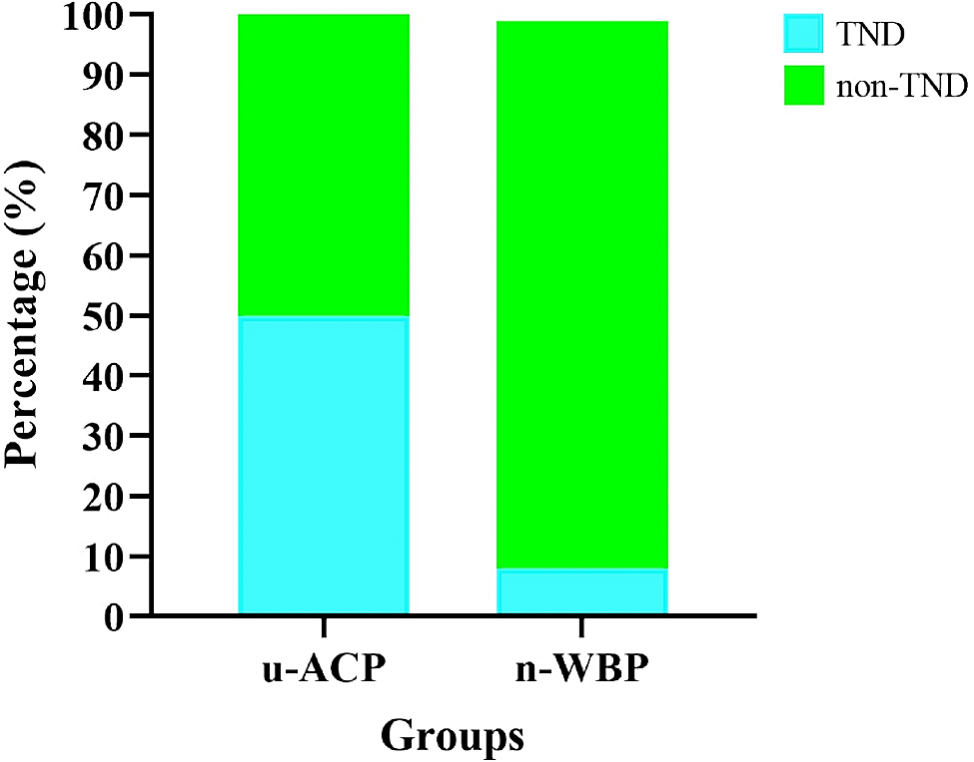
Bar diagram of incidence of TND

There were no severe complications, such as PND, ARF or death, in either group (Table 2). In the n-WBP group, there was 1 case of TND, with the patient experiencing agitation after awakening. In the u-ACP group, there were 9 cases of TND: 3 involving delirium and 6 involving agitation. Compared with patients in the u-ACP group, those in the n-WBP group had shorter VART [28.38 ± 12.77 vs. 59.97 ± 32.77 h, *P* = 0.017] and CICU stays [72.00 (66.00) vs. 108.00 (78.00) h, *P* = 0.038] (Fig. 11) and a lower incidence of TND [8.33 vs. 50.00%, *P* = 0.024) (Fig. 12).

### Follow-up results

At 1 year after surgery, there were no deaths in either group. Twenty-eight patients achieved good postoperative recovery without chest or back pain, fatigue or other symptoms. One patient in each group had persistent dull chest and back pain after surgery, which resolved spontaneously at 3 months. No patients had obvious abnormalities, such as aortic valve insufficiency, aortic root or DAO dilatation, as identified by TTE and CTA, or secondary operations.

## Discussion

### Safety of the branch-first approach with a novel continuous WBP strategy

u-ACP is safe and effective. However, the longer CPB, ACC and HCA durations observed in the u-ACP group could have some potential impacts [12–15]. To minimize adverse effects and improve surgical safety, our center innovatively adopted a branch-first approach with a n-WBP strategy for total arch surgery, achieving good surgical outcomes. Prioritized reconstruction of SAVs is a prerequisite for the n-WBP strategy. Therefore, we call this new technique the branch-first approach with a n-WBP strategy. Theoretically, the n-WBP strategy could be used in patients with serious aortic dilatation or effusion. Even in patients with severe aortic incompetence, rhythm and blood pressure can be preserved with the n-WBP during reconstruction of SAVs. Therefore, neither aortic incompetence nor AAO dilatation prevents the reconstruction of SAVs. It is important to cannulate the uninvolved IA or LCCA by dissection to increase the safety of the procedure.

In this study, surgery was successful in all 12 patients in the n-WBP group. Because arch anastomoses between the LCCA and LSCA salvages the area of the recurrent nerve, no recurrent nerve paralysis occurred in any of the patients after surgery. Moreover, there were no significant differences between the two groups in terms of the incidence of postoperative complications, such as AFR and death. However, the CPB, ACC and HCA durations were significantly shorter in the n-WBP group than in the u-ACP group. These findings suggested that the n-WBP strategy may have a reduced impact on organs and prevent damage to the myocardium associated with prolonged CPB and ACC durations [16, 17]. In addition, the ventilator-assisted respiration time and CICU stay were significantly shorter in the n-WBP group than in the u-ACP group. This may reduce the risk of pulmonary infection [18, 19] and reduce physical, mental, and economic burdens on patients. More importantly, our aim in adopting this new technique was to prioritize the blood supply to the brain, providing cerebral protection during procedures such as proximal and distal aortic anastomosis [20]. Fortunately, the new technique did not increase the incidence of postoperative complications or mortality in this study.

### Cerebral protective effects of the branch-first approach with a novel continuous WBP strategy

Total arch surgery is typically an emergency procedure, especially for patients with ATAAD, and is associated with postoperative complications and high mortality. Cerebral protection is crucial when performing TAR in response to ATAAD. There is a potential risk of brain injury regardless of whether the cerebral protection strategy involves deep HCA, u-ACP or b-ACP [21, 22]. Therefore, if physiologic WBP can be achieved, this risk could be reduced [23, 24]. For u-ACP, it is necessary to interrupt the blood supply to the LCCA and LSCA while they are anastomosed to the branches of the VG [25]. However, the n-WBP strategy does not interrupt the cerebral blood supply during anastomosis of the SAVs. In addition, we believe that continuous WBP may theoretically enhance spinal cord protection [26].

In this study, there were no significant differences in cerebral oxygenation during cerebral perfusion or in the incidence of postoperative PND between the two groups. However, the patients in the n-WBP group had a lower incidence of postoperative TND than those in the u-ACP group. The n-WBP strategy more closely mimics physiological cerebral perfusion than u-ACP. The incidence of TND in the u-ACP group was greater than that in the n-WBP group. There may be multiple reasons for this, including excessive cerebral perfusion, which results in brain tissue edema, leading to temporary and reversible brain injury. The difference in the incidence of TND between the two groups in this study suggested that the branch-first approach with a n-WBP strategy has certain application value for cerebral protection.

### Protective effect of the branch-first approach with a novel continuous WBP strategy on coagulation function

Initially, we typically used pure deep hypothermia to interrupt circulation for cerebral protection. However, in deep hypothermia, blood tends to be in a diluted state, with reduced platelet and coagulation factor concentrations and more blood seepage [27]. In addition, deep hypothermia may inhibit the function of thrombin in the coagulation cascade, leading to coagulation dysfunction, increased intraoperative bleeding and CNS complications [28]. To alleviate these adverse effects, we used u-ACP or b-ACP, which involve moderate hypothermia at 25–28 °C during circulatory arrest, to reduce the adverse effects of deep hypothermia. However, longer HCA and CPB durations also have some negative impacts on coagulation function.

In this study, compared with the patients in the u-ACP group, those in the n-WBP group had shorter HCA and CPB durations. The patients in the n-WBP group also had fewer amount of erythrocytes and plasma transfused. This may have been related to the shorter HCA and CPB durations. It indirectly suggests that the new technique may have the protective effect on coagulation function.

### Advantages and disadvantages of the branch-first approach with a novel continuous WBP strategy

The advantages of the new technique are as follows: (1) In the n-WBP method, the cerebral blood supply is not interrupted when the SAVs are anastomosed, while the u-ACP method requires clamping of the SAVs. (2) During anastomosis of SAVs, the n-WBP method allows cerebral blood supply via heart ejection, whereas the u-ACP relies on CPB to maintain cerebral blood supply. (3) Compared with u-ACP, n-WBP more closely mimics physiological cerebral perfusion. However, this new technique has several disadvantages. The greatest disadvantage of this approach is that additional procedures and tubes are needed. In addition, the risk of adverse cerebral perfusion may increase when dissection extensively involves the SAVs.

### Research strengths and limitations

This study reveals the value of this new technique for cerebral protection during arch surgery and provides a theoretical basis for subsequent studies. Additionally, the description of the new technique in this study is informative for future clinical practice related to total arch surgery. This study has several limitations. This was a single-center retrospective cohort study. The main limitations were the small sample size and possible selection and recall bias. In addition, although there were no statistically significant differences in baseline data (including surgical methods) between the two groups, the data may still be unbalanced owing to the presence of confounding factors such as uncollected characteristics, which may have affected the study results.

## Conclusions

In this study, the branch-first approach with a n-WBP strategy had no obvious disadvantages compared with u-ACP in terms of cerebral protection and surgical safety. These findings suggest that this new technique is feasible and has application value for ATAAD surgery.

## Data Availability

The data underlying this article will be shared on reasonable request to the corresponding author.
